# Supplementary material for: Optimized protocols for RNA interference in Macrostomum lignano
Source: G3 (Bethesda). 2024 Feb 29;14(5):jkae037. doi: 10.1093/g3journal/jkae037 (PMC11075559; doi:10.1093/g3journal/jkae037)
Supplement: jkae037_Supplementary_Data [file jkae037_supplementary_data.zip › Supplemental_Table_S1_G3-2023-404704.pdf]

Supplementary Table S1

Primers	Forward (5'-3')	Reverse (5'-3')
Primers for Topo-PCR-4-TA and pGEM-T cloning systems		
Mlig-ddx39b	GGGTGAACATCAGCGTGTAC	TCCTCTTCTCGAAGTTCTTG
Mlig-sperm1	TAGCCCTGCTAAGGCAGGTA	CGATGTTGGCGTTTCTTCTT
Mlig-piwi	TGCTCAAGCTGGTGTTGC	GTCTTGTTGTTGTGCCGC
gfp	CGTAAACGGCCACAAGTTCAG	GAACTCCAGCAGGACCATGTG
Mlig-tim29	AAAGTTGAAAGCAAATTTCTGG	GCCAATCGATATAAAATTGGAA
pGEM-T	CGGCCGCCATGGCCGCGGGA	TGCAGGCGGCCGCACTAGTG
pGEM-T with T7 extension	ggatcctaatacgactcactatagg CGGCCGCCATGGCCGCGGGA	ggatcctaatacgactcactatagg TGCAGGCGGCCGCACTAGTG
Primers for in vitro dsRNA synthesis. T7 promotors are in lower case		
piwi T7	taatacgactcactatagggaga TGCTCAAGCTGGTGTTGC	taatacgactcactatagggaga GTCTTGTTGTTGTGCCGC
ddx39b T7	taatacgactcactatagggaga GGGTGAACATCAGCGTGTAC	taatacgactcactatagggaga TCCTCTTCTCGAAGTTCTTG
sperm1 T7	taatacgactcactatagggaga TAGCCCTGCTAAGGCAGGTA	taatacgactcactatagggaga CGATGTTGGCGTTTCTTCTT
gfp T7	taatacgactcactatagggaga CGTAAAC	taatacgactcactatagggaga GAACTCC
Primers used to PCR amplify fragments to clone into T444T vector for bacterial production of dsRNA. Overhangs (oh) to T444T are in lowercase		
ddx39b-ohT444T	actatagggcgaattgggta TGCATGAACTTCTTGCAGATTG	actatagggcgaattgggta TGCATGAACTTCTTGCAGATTG
mNG-ohT444T	actatagggcgaattgggta ACGTCGGTGAAGGCCTTCT	actatagggcgaattgggta ACGTCGGTGAAGGCCTTCT
mSC-ohT444T	actatagggcgaattgggta ACGGTGTAGTCCTCGTTGT	actatagggcgaattgggta ACGGTGTAGTCCTCGTTGT
piwi-ohT444T	actatagggcgaattgggta GTCGCCGTTCTTCTCTCTGA	actatagggcgaattgggta GTCGCCGTTCTTCTCTCTGA
heh1-ohT444T	actatagggcgaattgggta CTGCTGGATGATTCTCGGTGA	actatagggcgaattgggta CTGCTGGATGATTCTCGGTGA
Primers for qPCR		
ribosomal S19	GTCTAACCAATGGCTGACGACC	TCGGAGAACTGATCGATGCTCATG
piwi	AGGCCATTGTGGTGAAGAAG	ACTGCGACACCAGGAAGAAG
sperm1	GGCCGGCGGGGTGTATGA	AGTTGGCTGCAGCTCCGTCTCC
ddx39b	TTCACGGACTCAAGCAGCACTACCT	CCAGAACGTCGATCAGCTCAAACAG
