# Supplementary figures and images for: Optimized protocols for RNA interference in Macrostomum lignano
Source: G3 (Bethesda). 2024 Feb 29;14(5):jkae037. doi: 10.1093/g3journal/jkae037 (PMC11075559; doi:10.1093/g3journal/jkae037)

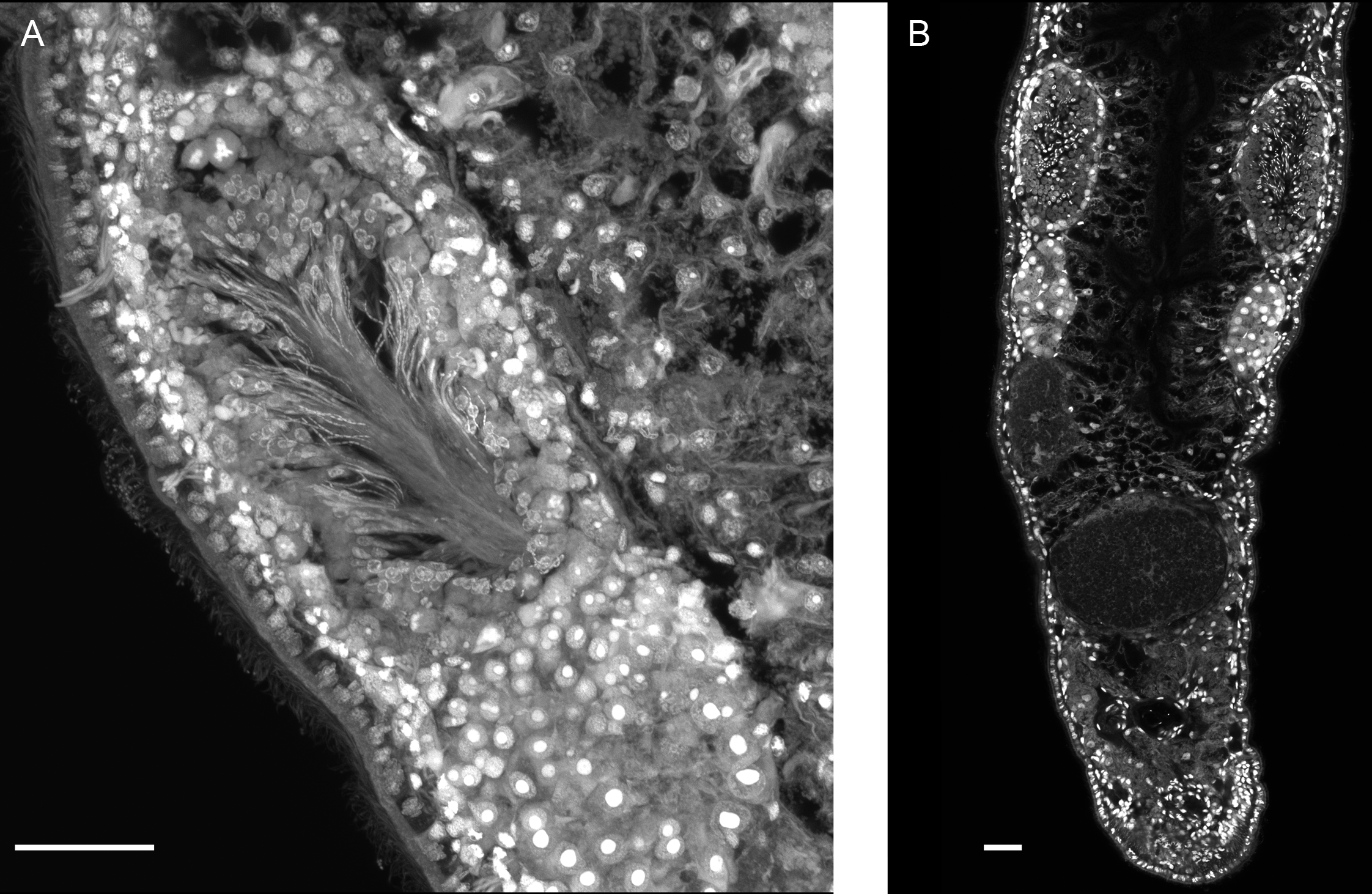

Supplement: jkae037_Supplementary_Data [file jkae037_supplementary_data.zip › Supplemental_Figure_S1_G3-2023-404704.tif]
